# Characterization of *Listeria monocytogenes* strains isolated from soil under organic carrot farming

**DOI:** 10.3389/fmicb.2024.1530446

**Published:** 2025-01-21

**Authors:** Maciej Nowak, Zbigniew Paluszak, Natalia Wiktorczyk-Kapischke, Katarzyna Grudlewska-Buda, Anna Budzyńska, Joanna Skonieczna-Kurpiel, Ewa Wałecka-Zacharska, Monika Huse-Kutowska, Krzysztof Skowron

**Affiliations:** ^1^Department of Microbiology and Food Technology, Bydgoszcz University of Science and Technology, Bydgoszcz, Poland; ^2^Department of Microbiology, Collegium Medicum of L. Rydygier in Bydgoszcz, Nicolaus Copernicus University in Toruń, Bydgoszcz, Poland; ^3^Department of Food Hygiene and Consumer Health Protection, Wrocław University of Environmental and Life Sciences, Wrocław, Poland

**Keywords:** *Listeria monocytogenes*, organic farming, soil, biofilm, metabolic rate, invasiveness, antibiotic resistance

## Abstract

**Introduction:**

*Listeria monocytogenes* are Gram-positive, non-spore-producing rods that are the etiological agent of listeriosis. *L. monocytogenes* is isolated from soil, water, sewage, rotting vegetation, and the main source of these rods for humans is food (fish, unpasteurized dairy products, or raw fruits and vegetables). In recent years, there has been an increase in consumer interest in healthy food, especially organically grown. The use of natural fertilizers during organic farming can be a source of pathogens, including *L. monocytogenes* in the soil and finally in vegetables and fruits. The aim of this study was to assess the prevalence of *L. monocytogenes* in soil samples from organic carrot crops (Poland) and to characterize the tested strains. Microbial contamination of the soil has a direct impact on the safety of the crops grown on it. This is an important aspect in organic farming, where products are chosen as a healthier option and consumed by children and the elderly.

**Methods:**

The isolates were subjected to genetic similarity assessment (PFGE method), and the tested strains were evaluated for antibiotic susceptibility (disc-diffusion method), invasiveness (HT-29 line human colon cancer cell line), coaggregation with *Salmonella* Enteritidis, biofilm-forming ability and the effect of disinfectants on the biofilm.

**Results:**

Twenty-seven isolates of *L. monocytogenes* were isolated from 250 soil samples, 10 of which were genetically different. 80% of the tested strains were sensitive to the tested antibiotics. Antibiotic resistance was demonstrated in two strains (strain 11 – resistant to penicillin and cotrimoxazole, strain 22 – resistant to penicillin). The highest invasiveness against HT-29 cells at 23.2% was shown for strain 11. However, invasiveness of less than 1% was demonstrated for three strains, and strain 13 showed no ability to invade HT-29 human colon cancer cells. The level of coaggregation between the tested strains and *S.* Enteritidis ranged from 22.2 to 39.1%. The number of biofilm-isolated rods from the stainless steel surface was 6.37 to 7.10 log colony-forming unit (CFU)/cm^2^, while on polypropylene it was from 6.75 to 8.06 log CFU/cm^2^. The effectiveness of the disinfectants used depended on the duration of action and the concentration of the disinfectant. Chlorosol was shown to be the disinfectant causing stronger biofilm eradication on each of the tested surfaces. It has been shown that soils and thus food from organic farming can be a source of *L. monocytogenes*. These rods can vary in phenotypic characteristics and virulence levels.

**Discussion:**

The research conducted allows to draw attention to the occurrence of pathogens, including *L. monocytogenes* in crops from organic farming. In addition, the results presented can help to introduce standards regulating the safety of organic farming, taking into account the occurrence of antibiotic-resistant or highly invasive strains, thus maintaining food safety.

## 1 Introduction

Agriculture, is a fundamental source of food products, essential for the entire society. In recent years, there has been a noticeable increase in the promotion of organic farming, due to its numerous environmental benefits, as well as the benefits of future consumers making an informed choice of healthy food products (Scialabba and Hattam, 2002). The principle of organic farming is to refrain from using agricultural, veterinary and food chemicals in the food production process (Dumontet et al., 2017). What results is efficient production that combines practices that promote environmental protection and preserve access to limited natural resources (Dumontet et al., 2017).

Despite the many benefits of organic farming, it is important to remember the food safety risks of using natural fertilizers. Contamination of vegetables can occur during cultivation, harvesting or distribution (Mandrell, 2009). An important element of organic farming is soil. According to the Regulation of the European Parliament and of the Council (EU) 2018/848 (2018), soil fertility and biological activity are maintained and increased, mainly through the use of manure, or organic matter. In manure or slurry, under the right conditions, pathogenic bacteria can survive for several weeks or even months and then enter the soil, water or crops causing contamination (Alegbeleye et al., 2018). It has been shown that the greater the contact of plants with contaminated soil, the greater the degree of contamination, including microbial. Fertilizers have been shown to be reservoirs of enteric pathogens that can contaminate crops, fruits and vegetables (Jiang and Shepherd, 2009). A higher risk of contamination applies to vegetables and fruits in direct contact with the soil, such as root and leafy vegetables (e.g., carrots) (Islam et al., 2004). Another important source of microbial contamination of the soil environment is water. Agriculture mostly uses ground and surface water, into which biological agents can penetrate under certain conditions. The main risks are spills from reservoirs or manure storage sites, livestock and wildlife feces, or water runoff from contaminated fields (Alegbeleye et al., 2018). Numerous bacterial pathogens are well adapted to survive and multiply in both soil and water. These microorganisms usually form a biofilm that adheres to the plant surface, or migrate through the root system to other plant parts (Ryser et al., 2009; Yaron, 2014). The process of washing fruits and vegetables only removes surface contaminants, without removing the biofilm crust, or pathogens present in the inner tissues of the plants (Vogeleer et al., 2014). Numerous reports on organically grown vegetables (Heaton and Jones, 2008; Maffei et al., 2013; Tango et al., 2014), have shown that consuming food products from such farming does not increase the incidence of foodborne illness. Therefore, the issue of microbiological safety of organic food is constantly questioned (Maffei et al., 2016). However, it has been shown that raw vegetables, including those from organic farming, can be contaminated by pathogens such as, *Listeria monocytogenes*, *Salmonella* Typhimurium, *Escherichia coli*, *Campylobacter* spp. (Golberg et al., 2011; Kljujev et al., 2018).

One of the more significant pathogen risks associated with organic farming is *L. monocytogenes. L. monocytogenes* are Gram-positive, non-spore-producing rods characterized by adaptation to variable and adverse environmental conditions (Wiktorczyk-Kapischke et al., 2023). The main source of *L. monocytogenes* for humans is food, mainly products from unpasteurized milk, meat and meat products, fish and fishery products, and raw fruits and vegetables (European Food Safety Authority and European Centre for Disease Prevention and Control, 2023). It should be emphasized that listeriosis has a high mortality rate, at around 30%. The most vulnerable to infection are the elderly, pregnant women, newborns, or immunocompromised people (World Health Organization, 2018). The source of contamination of vegetable products by *L. monocytogenes* can be soil, manure and water (Kljujev et al., 2018). Some studies have shown that *L. monocytogenes*, can survive on the surface of a damaged seed coat during plant germination. This implies the possibility of *L. monocytogenes* contamination of the entire plant (Gorski et al., 2004). A key aspect affecting the problems of eliminating *L. monocytogenes* from raw fruits and vegetables is the ability of these rods to form a biofilm (Oliveira et al., 2010). According to Botticella et al. (2013), biofilm formation allows *L. monocytogenes* to persist for long periods of time in the food processing environment, and therefore is a source of recurrent contamination and poses a food safety risk. Due to the increasing number of patients diagnosed with listeriosis and the identification of multidrug-resistant strains, it is advisable to assess antimicrobial resistance among *L. monocytogenes* isolates from food products (European Food Safety Authority and European Centre for Disease Prevention and Control, 2023). Thus, it is important to assess the occurrence of *L. monocytogenes* in soil from organic crops in order to assess food safety risks.

The aim of this study was to evaluate the occurrence of *L. monocytogenes* in soil samples from organic carrot cultivation (Poland) and to characterize selected features of the obtained strains determining their virulence. The above objective is particularly significant because microbial contamination of soil directly affects crop safety, especially in organic farming, where produce is often selected for its health benefits and consumed by vulnerable groups like children and the elderly.

## 2 Materials and methods

### 2.1 Material

#### 2.1.1 Soil sampling and isolation of *Listeria monocytogenes*

The material for the study consisted of 250 soil samples taken from 5 fields (Poland) where carrots were grown organically. The cultivation was carried out on light sandy loam soils classified as quality class IVa. The preceding crops were cucumbers or onions. Organic farming practices had been implemented on these fields for 5 years. The growing season lasted approximately 220 days. Soil samples were taken from sites in the immediate vicinity of the growing carrots. Fifty samples of 200 g per field were taken.

After delivery to the laboratory, samples were shaken in sterile buffered saline (PBS, Sigma-Aldrich) for 24 h (300 rpm, room temperature) and then sonicated for 15 min (Ultrasonic DU-4, Nickel-Electro Ltd., United Kingdom).

Then, 25 mL of the resulting suspension was transferred to 225 mL of half-Fraser broth (Oxoid) and subjected to pregrowth for *L. monocytogenes*. After 24 h incubation (30°C), 1 mL of the culture was transferred to 9 mL of Fraser broth (Oxoid) and incubated (37°C, 48 h). After the secondary multiplication step, reduction culture was performed on medium according to Ottaviani and Agosti (ALOA) (Oxoid) and incubated (24 h, 37°C). The grown colonies, typical of *L. monocytogenes*, were screened on Columbia Agar with 5% sheep blood (Biomerieux) and incubated (37°C, 24 h). The grown colonies were used for further studies.

### 2.2 Methods

#### 2.2.1 Identification of *Listeria monocytogenes* strains using the PCR method

Species identification of the strains was carried out by PCR using previously isolated DNA. DNA isolation was performed using the Genomic Mini kit (A&A Biotechnology) according to the manufacturer’s procedure. The primer pair L1 (5’-CAG CAG CCG CGG TAA TAC-3′) and L2 (5’-CTC CAT AAA GGT GAC CCT-3′) (product size: 938 bp) (Border et al., 1990), designed based on the 16S rRNA sequence allowed the evaluation of the affiliation of the tested strains to the genus *Listeria*, while the pair LM1 (5’-CCT AAG ACG CCA ATC GAA-3′) and LM2 (5′-AAG CAC TTG CAA CTG CTC-3′) (product size: 750 bp) (Bansal, 1996), designed based on the sequence of the gene encoding listeriolysin O (LLO) allowed the identification of isolates to the species *L. monocytogenes*.

The standardized PCR protocol for 25 μL reaction mixture included 1 × PCR buffer (Promega), 2 mM MgCl_2_ (ABO), 1.25 mmol dNTPs (Promega), 0.5 μM of each primer (Oligo.pl), 1 unit of Taq DNA polymerase (Promega) and ultrapure water. DNA isolated from *L. monocytogenes* ATCC 19111 strain was the control. The PCR program was set as follows: initial denaturation 94°C/2 min; 30 cycles of denaturation 94°C/30 s, annealing 50°C/30 s and duration 72°C/1 min; extension 72°C/1 min.

#### 2.2.2 Assessment of genetic similarity of isolates using PFGE technique

The genetic similarity of the isolates was assessed using pulsed-field gel electrophoresis (PFGE), a procedure carried out in accordance with PulseNet recommendations (PulseNet USA, 2013). Bacterial suspensions were prepared in TE buffer (10 mM Tris–HCl, 100 mM EDTA) (Novazyme) with a MacFarland density of 4.0. Then, lysozyme (10 mg/mL, EurX) and proteinase K (20 mg/mL, Thermo Fisher Scientific) were added to the suspension and incubated (55°C, 40 min). Blocs were prepared using 1.0% agarose (Certified Megabase) (Bio-Rad). After solidification, the blocks were incubated (54°C, 2 h) in lysis buffer (50 mM Tris–HCl, 50 mM EDTA, 1.0% lauryl sarcosyl (Sigma-Aldrich), 0.15 mg/mL proteinase K). Then, the blocks were washed twice in ultrapure water and four times in TE buffer. Pre-restriction was carried out at 30°C for 10 min. DNA restriction (30°C, 7 h) was carried out in buffer consisting of: *ApaI* enzyme (10 U/μl) (Thermo Fisher Scientific), Tango buffer (Thermo Fisher Scientific), water. Electrophoretic separation was carried out in a 1.0% agarose gel (Certified Megabase) (Bio-Rad) in a CHEF Mapper apparatus (Bio-Rad) using the following electrophoresis conditions: initial and final pulse duration: 4–40 s, voltage: 6 V/cm, pulse angle: 120°, temperature: 14°C. Electrophoresis was carried out for 17 h. Image visualization was performed using the GelDoc XR system (Bio-Rad).

To determine the degree of genetic similarity between the strains studied, a phylogenetic dendrogram was plotted using CLIQS 1D Pro software (TotalLab). Clustering analysis was performed using hierarchical clustering using the Unweighted Pair Group Method of Aritmetic Means (UPGMA) technique with Dice coefficient.

#### 2.2.3 Assessment of antibiotic susceptibility

The susceptibility of *L. monocytogenes* strains to antibiotics was assessed using the disc-diffusion method on Mueller-Hinton Agar with 5.0% horse blood and 20 mg/L *β*-NAD (MHF) (bioMérieux).

The tested strains were cultured from freezing onto CAB medium. After 20 h of incubation, a suspension of bacteria in 0.9% NaCl (Avantor) at a density of 0.5 McFarland was prepared. Inoculum was spread on MHF medium, then antibiotic discs [penicillin (1 U), ampicillin (2 μg), meropenem (10 μg), erythromycin (15 μg), trimethoprim-sulfamethoxazole (1.25–23.75 μg)] (Argenta) were applied. The performed antibiograms were incubated at 35°C for 20 h. After the incubation period, zones of growth inhibition around the discs were measured and the results were interpreted according to the recommendations of EUCAST v. 12.0 (European Committee on Antimicrobial Susceptibility Testing, n.d).

#### 2.2.4 Evaluation of the invasiveness of *Listeria monocytogenes*—plaque forming assay test

Single colonies were transferred to 5 mL of Brain Heart Infusion (BHI) broth and incubated in a thermoblock (TDB-100, Biosan) at 37°C (230 rpm, 6 h). In the next step, 5 μL of the bacterial suspension was transferred to 5 mL of BHI broth and incubated 18 h until an OD_600_ of 2.4–2.6 was obtained (measured with a DU 8800D spectrophotometer). Multiplied bacteria, at 5–6 log CFU, were used to infect HT-29 human colon cancer cell line.

HT-29 cells were seeded into 6-well polystyrene culture plates (Genoplast) and incubated until approximately 90% confluence in Dulbecco’s Modified Eagle Medium (DMEM) (Sigma-Aldrich), formulated with: 10% FBS (fetal bovine serum) (Gibco), 2 mM glutamine, and 100 IU/mL penicillin and 100 μg/mL streptomycin (Sigma-Aldrich). Then, 24 h before the infection was performed, the medium was changed to DMEM containing no antibiotics. A suspension of bacteria in BHI at 5–6 log CFU was added to HT-29 cell cultures and incubated for 2 h (37°C, 5% CO_2_). The wells were then washed twice with sterile PBS solution (Sigma-Aldrich), the medium was changed to DMEM containing 100 μg/mL gentamicin (Sigma-Aldrich) and incubated for 1.5 h (37°C, 5% CO_2_). The gentamicin medium was removed, and medium containing 10 μg/mL gentamicin and 1.0% low-melting point agarose (Prona, Gdansk) was added to the wells. After 48 h, the number of plaques was determined. Bacterial invasiveness was expressed as a quotient of the number of plaques, expressing the number of bacteria that penetrated into HT-29 cells, and the number of bacteria entering the wells. Invasiveness was expressed as a percentage.

#### 2.2.5 Evaluation of the metabolic rate of *Listeria monocytogenes* strains

From the grown colonies of each *L. monocytogenes* strain, a 0.5 McF suspension was prepared in sterile PBS solution (Sigma-Aldrich). The suspensions were then diluted 100-fold in tryptose-soy broth (TSB) (Becton Dickinson).

A set of two plates was prepared: (1) containing tetrazolium salt (MTT) (Sigma-Aldrich); (2) without it. Into the wells of the multiwell plates (set with MTT), 80 μL of TSB was introduced, followed by the addition of 20 μL of MTT solution (5 ng/mL) and 100 μL of suspension (3 replicates). In the set-up without MTT, plates contained 100 μL of TSB and 100 μL of suspension of each strain (3 replicates). The negative control was 200 μL of sterile TSB (3 replicates).

The plates were incubated at 37°C in a humid chamber. After a predetermined incubation time (0, 1, 2, 3, 4, 5, 7, and 24 h), absorbance was measured using a Synergy HT (BIO-TEK) multi-detector microplate reader at 570 nm. After the designated incubation time, a solution of acidic isopropanol (isopropanol (Avantor) + 5% (v/v) 1 M HCl (Avantor)) with a volume of 200 μL and a concentration of 0.04 mol/dm3 was added to each well of the kit with MTT to dissolve the precipitated formazone.

Absorbance of samples without the addition of MTT (to determine the multiplication of bacteria in suspension in TSB) was performed at 595 nm. No acidic isopanol solution was added to the plates and the same plates were further incubated after the measurement.

Immediately before the absorbance measurement, the plates were shaken for 15 min (300 rpm, room temperature). To determine the metabolic activity coefficient (MAC), the metabolic activity values (MAV) of a given strain at a specific measurement date were calculated according to the formula:


$$MAV=AMTT_{t}-Az_{t.}$$

where:

MAV, metabolic activity of a given strain from the test or control group after a specified incubation time t

A_MTTt_, absorbance value measured after a specified incubation time t of a given strain from the test or control group in a sample containing a bacterial suspension with MTT

A_zt_, absorbance value measured after a specified incubation time t of a given strain of the test and control group in a sample containing a bacterial suspension without MTT

Based on the metabolic activity, the metabolic activity coefficient of the strain was calculated, according to the formula:


$$MAF=\frac{\sum AM_{t}}{n}$$

where:

MAF, metabolic activity coefficient

∑AMt, sum of metabolic activity values from individual times (after 0, 1, 2, 3, 4, 5, 7 and 24 h of incubation) of a given strain from the control or test group

n, number of absorbance measurements made – 8

The value of the metabolic rate allowed us to compare the metabolic rate of the different *L. monocytogenes* strains tested.

#### 2.2.6 Assessment of coaggregation ability between *Listeria monocytogenes* and *Salmonella* Enteritidis

In this stage of the study, in addition to the obtained strains of *L. monocytogenes*, a strain of *S.* Enteritidis isolated from poultry meat was used. Based on 24 h cultures of *L. monocytogenes* and *S*. Enteritidis strains on CAB medium, suspensions of the tested isolates were made at an optical density of 0.7 McF in reaction buffer (1.0 mM Tris–HCl (Sigma Aldrich), 0.1 mM MgCl_2_ (Avantor), 0.1 mM CaCl_2_ (Avantor), 0.15 M NaCl (Avantor)) (Kinder and Holt, 1994). A suspension of *L. monocytogenes* test isolates (1 mL) was combined with a suspension of *S*. Enteritidis (1 mL) and incubated at 37°C for 2 h. At the same time, an autoaggregation test was performed, in which 1 mL of *L. monocytogenes* suspension and 1 mL of *S.* Enteritidis suspension in reaction buffer were incubated (2 h, 37°C) in separate tubes. After the incubation period, the suspensions were subjected to centrifugation (7 × g, 2 min.), and the supernatant (0.6 mL) was collected and analyzed spectrophotometrically (Beckman DU-640 spectrophotometer) at 650 nm (Kinder and Holt, 1994). The assessment of coaggregation levels for species X (*L. monocytogenes*) and Y (*S.* Enteritidis) was calculated according to the formula:


$$\begin{array}{l} \%\mathrm{coaggregation}\\ =\left[\left(\left[\left\{A650X+A650Y\right\}/2\right]–A650\;\left(X+Y\right)\right)/\left\{A650X+A650Y\right\}/2\right]\;x\;100 \end{array}$$

where:

% coaggregation, the level of coaggregation between strains

A_650_, absorbance value at 650 nm

X, autoaggregation level of strain X (*L. monocytogenes*)

Y, autoaggregation level of strain Y (*S.* Enteritidis)

X + Y, coaggregation level of strain X (*L. monocytogenes*) and Y (*S.* Enteritidis) (Kinder and Holt, 1994)

#### 2.2.7 Assessment of biofilm formation

The ability to form biofilm on sterile polypropylene and AISI 304 stainless steel fragments (1cm^2^ area) (washed and sterilized) was evaluated. Test strains were cultured on CAB medium and, after incubation (24 h, 37°C), 3 mL suspensions of test strains at a density of 0.5 McF in BHI medium were prepared. Sterile polypropylene or stainless steel fragments were placed in the suspensions and incubated (24 h, 37°C). After this time, the stainless steel or polypropylene fragments were rinsed in sterile PBS solution and transferred to sterile BHI medium. The procedure was repeated 2 times. Then, the surfaces were rinsed twice in PBS solution and sonicated in an Ultrasonic DU-4 sonicator (Nickel-Electro) for 20 min. After sonication, a series of decimal dilutions were made in PBS solution to a dilution of 10^−6^ and cultures at 0.1 mL per CAB medium. The plates were incubated for 24 h at 37°C. After incubation, the grown colonies were counted, and the result was reported as the number of colony-forming units (CFU) per cm^2^ of test area (CFU/cm^2^).

#### 2.2.8 Effectiveness of disinfectants on biofilm formed *Listeria monocytogenes*

The biofilm produced on stainless steel and polypropylene was used to evaluate the effectiveness of two disinfectants:

1. Alusol (an aqueous solution of phosphoric acid, hydrochloric acid and non-ionic surfactants) (Radex)
2. Chlorosol (an aqueous solution of sodium hypochlorite and stabilizing substances) (Radex)

Working solutions were prepared on the basis of sterile hard water (Polish Standard PNEN-1276, 2010) at concentrations of: 0.1, 0.5 and 1.0%. Test surfaces with biofilm formation were immersed in the respective concentrations of disinfectant solutions for 1, 5 and 15 min. After this time, the samples were shaken for 2 min (400 rpm) in a neutralizer (tween 80 (Sigma-Aldrich) - 10.0 g; lecithin (Sigma-Aldrich) - 1.0 g; histidine L (Sigma-Aldrich) - 0.5 g; Na_2_S_2_O_3_ (Avantor) - 2.5 g; water - 1000 mL) and sonicated in a sonicator for 10 min. After sonication, a series of decimal dilutions were made (up to 10^−6^ in PBS) and cultured at 0.1 mL per CAB medium. Plates were incubated for 24 h at 37°C. After incubation, the grown microbial colonies were counted and reported as CFU/cm^2^ of the area tested.

The negative control consisted of stainless steel and polypropylene fragments incubated in sterile BHI solution under the same conditions.

#### 2.2.9 LIVE/DEAD fluorescence staining

For the tested *L. monocytogenes* strains, stained slides were prepared using the LIVE/DEAD Bac Light Bacterial Viability Kit (Thermo Fisher Scientific). In these preparations, the proportion of live and dead *L. monocytogenes* cells in a single layer of biofilm formed on the surface of stainless steel and polypropylene, treated and untreated with disinfectants, was determined under a fluorescence microscope (Nikon Eclipse Ci, magnification 1,000x).

Samples were stained with the LIVE/DEAD Bac Light Bacterial Viability Kit according to the manufacturer’s instructions and incubated for 15 min at 37°C without light.

#### 2.2.10 Statistical analysis

The results for biofilm formation on the tested surfaces and the effectiveness of the disinfectants used were converted to logarithms (log CFU). When evaluating the effectiveness of disinfectants, logarithmic decreases in the number of bacteria were calculated.

Means were calculated for the results obtained. Based on Statistica (TIBCO Software Inc., Palo Alto, CA, USA) software checked for the occurrence of significant differences in the strength of biofilm formation depending on the strain of *L. monocytogenes* and the tested surface. The existence of statistically significant differences between decreases in the number of bacteria recovered from the biofilm under disinfection depending on the disinfectant used, its concentration and duration of action, and the type of surface was also checked. A multivariate analysis of variance ANOVA was performed in both cases and Tukey’s post-hoc test was used with a significance level of 0.05.

## 3 Results

### 3.1 Prevalence of *Listeria monocytogenes* in collected soil samples

The percentage of positive soil samples was 10.8% (Table 1). Twenty-seven isolates of *L. monocytogenes* were isolated (Table 1). Isolates of the tested rods were not obtained from samples from P2 and P5 fields. By multiplex PCR, it was confirmed that all isolates used in the study belonged to the *L. monocytogenes* species.

**Table 1:** Number of *L. monocytogenes* isolates obtained from the samples tested.

Voivodeship	Field	Number of samples	Number of isolates (percentage of positive samples)	Isolate number
Greater Poland (Poland)	P1	50	10 (20.0%)	1, 2, 3, 4, 5, 6, 7, 8, 9, 10
	P2	50	0 (0.0%)	---
Kuyavia-Pomerania (Poland)	P3	50	11 (22.0%)	11, 12, 13, 14, 15, 16, 17, 18, 19, 20, 21
	P4	50	6 (12.0%)	22, 23, 24, 25, 26, 27
	P5	50	0 (0.0%)	---
Total		250	27 (10.8%)	---

### 3.2 Evaluation of genetic similarity of tested isolates

**Figure 1:**
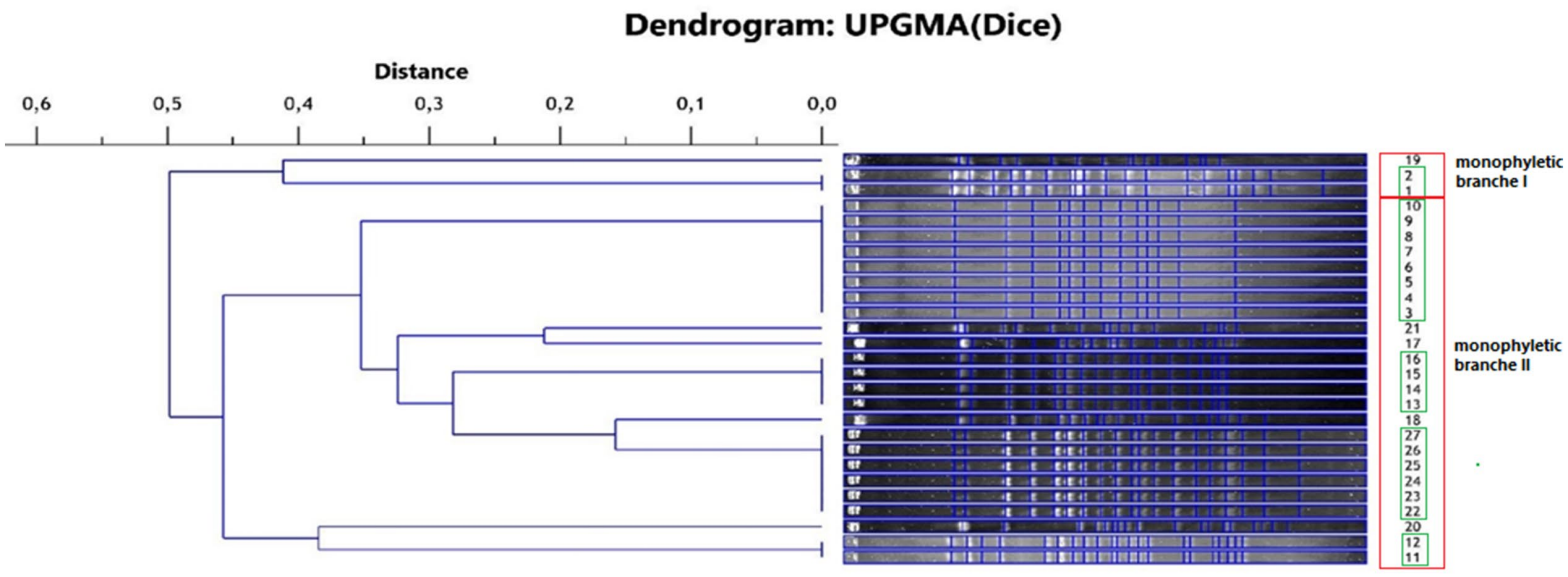
Phylogenetic dendrogram of the genetic similarity of the tested *L. monocytogenes* isolates (red frame—monophyletic branch, green frame—isolates representing the same strain).

The isolates of *L. monocytogenes* were classified into two main monophyletic branches (Figure 1). Branch I included 3 isolates from field P1 and P3, and branch II included 24 isolates from all fields with positive samples. Five groups comprising genetically identical isolates were shown. The first group included isolates: 1 and 2 from field P1, the second group included isolates 3–10 from field P1, the third group included isolates 11–12 from field P3, the fourth group included isolates 13–16 from field P3 and the fifth group included isolates 22–27 from field P4. The largest number of isolates (*n* = 11), and among them the largest number of genetically different strains (*n* = 5) were found in samples originating from field P4.

From the pool of 27 isolates, 10 genetically different strains of *L. monocytogenes* were isolated and used in further studies.

### 3.3 Assessment of antibiotic susceptibility of tested strains of *Listeria monocytogenes*

The susceptibility of the tested strains to five antibiotics (penicillin, ampicillin, meropenem, erythromycin, trimethoprim-sulfamethoxazole) was evaluated. It was found that 8 (80.0%) strains were susceptible to all antibiotics tested. One strain (10.0%) was resistant to penicillin and cotrimoxazole, and one (10.0%) strain was resistant to penicillin, with preserved susceptibility to other antibiotics.

### 3.4 Evaluation of the invasiveness of the tested strains of *Listeria monocytogenes*

The ability of *L. monocytogenes* to adhere and invade is directly related to the virulence level of this pathogen (Wałecka-Zacharska et al., 2013). Invasive form of listeriosis (especially among high-risk groups) is associated with a high mortality rate (World Health Organization, 2018). It was shown that the highest (23.22%) invasiveness was characterized by strain 11 and the lowest (0.38%) by strain 3 (Table 2). Strain 13 was not found to have the ability to invade HT-29 human colon cancer cells. Low invasiveness of less than 1% was characterized by three strains (3, 17 and 21), and high invasiveness of 17.77% was also shown for strain 22 (Table 2).

**Table 2:** Invasiveness of the tested strains of *L. monocytogenes.*

Strain number	Invasiveness [%]
1	3.09 ± 1.14
3	0.38 ± 0.12
11	23.22 ± 8.83
13	n.f.*
17	0.58 ± 0.10
18	3.11 ± 1.07
19	1.66 ± 0.31
20	1.46 ± 0.55
21	0.89 ± 0.06
22	17.77 ± 3.38

n.f.*, not found.

### 3.5 Evaluation of the metabolic rate of *Listeria monocytogenes* strains

The metabolic activity of the tested strains was examined using MTT. For the tested strains of *L. monocytogenes*, changes in metabolic activity were observed as the set time increased (Table 3). There was an increase in the absorbance of strains with and without MTT after the specified incubation time. The coefficient of metabolic activity in the test group (MTT) ranged from 0.748 for strain 17 to 1.117 for strain 19 (Table 3).

**Table 3:** The metabolic rate of the tested *L. monocytogenes* strains and the activity coefficient.

	MTT (absorbance 570 nm)										
	Strain number										
Time [h]	1	3	11	13	17	18	19	20	21	22	K(−)
0	0.200	0.213	0.199	0.192	0.163	0.201	0.225	0.194	0.173	0.186	0.161
1	0.461	0.529	0.493	0.479	0.516	0.399	0.614	0.357	0.617	0.351	0.184
2	0.801	0.826	0.801	0.907	0.651	0.701	0.896	0.768	0.749	0.715	0.176
3	1.156	1.185	1.123	1.052	0.867	1.006	1.201	1.191	0.967	1.154	0.169
4	1.199	1.294	1.369	1.170	1.115	1.131	1.400	1.412	1.238	1.312	0.203
5	1.492	1.732	1.701	1.599	1.434	1.502	1.867	1.526	1.591	1.720	0.129
7	2.137	2.287	2.215	2.001	1.701	1.806	2.315	1.973	1.909	1.962	0.191
24	2.788	2.951	2.873	2.618	2.104	2.241	3.019	2.259	2.296	2.206	0.188

	Without MTT (absorbance 595 nm)										
	Strain number										
Time [h]	1	3	11	13	17	18	19	20	21	22	K(−)
0	0.095	0.100	0.092	0.090	0.080	0.087	0.099	0.087	0.076	0.091	0.06
1	0.106	0.132	0.120	0.099	0.116	0.110	0.123	0.102	0.146	0.115	0.077
2	0.119	0.135	0.128	0.120	0.101	0.132	0.152	0.182	0.198	0.129	0.074
3	0.139	0.176	0.139	0.147	0.126	0.157	0.175	0.199	0.214	0.164	0.07
4	0.183	0.192	0.176	0.150	0.167	0.192	0.201	0.203	0.227	0.183	0.086
5	0.216	0.226	0.227	0.169	0.160	0.195	0.234	0.221	0.264	0.247	0.091
7	0.385	0.397	0.352	0.351	0.279	0.325	0.437	0.404	0.401	0.301	0.101
24	0.671	0.732	0.687	0.630	0.459	0.519	0.766	0.552	0.559	0.482	0.114

	Activity factor										
Strain number	1	3	11	13	17	18	19	20	21	22	
	0.900	1.070	1.058	0.992	0.748	0.934	1.117	0.894	0.949	0.840	

MTT, tetrazolium salt.

### 3.6 Evaluation of the ability to coaggregate between *Listeria monocytogenes* and *Salmonella* Enteritidis

Coaggregation is the process of reversible accumulation of bacterial cells of two different bacterial strains. Coaggregation plays an important role during surface colonization (including in processing plants or vegetable surfaces) and biofilm formation (Kinder and Holt, 1994). It was shown that the level of coaggregation between tested *L. monocytogenes* strains and *S.* Enteritidis ranged from 22.2 to 39.1% (Table 4). The highest (39.1%) level of coaggregation was found between *S.* Enteritidis and strain 21 (Table 4). In contrast, the lowest degree of coaggregation, at 22.2%, was shown between *S.* Enteritidis and strain no. 17 (Table 4).

**Table 4:** Coaggregation coefficient calculated for the tested strains of *L. monocytogenes.*

Strain number	Mix	Individually	Coaggregation [%]
1	0.031	0.039	35.4
3	0.039	0.051	27.8
11	0.038	0.044	24.8
13	0.036	0.056	36.3
17	0.042	0.051	22.2
18	0.036	0.052	33.9
19	0.038	0.052	30.3
20	0.031	0.036	33.3
21	0.039	0.071	39.1
22	0.038	0.056	32.7

### 3.7 Evaluation of biofilm formation on tested surfaces

One of the main causes of food contamination by *L. monocytogenes* is its ability to form a biofilm and survive in adverse environmental conditions. The multi-layered structure of the biofilm makes it difficult for biocides to reach the deeper layers of the biofilm. In this study, we evaluated the biofilm-forming ability of *L. monocytogenes* on the surface of stainless steel and polypropylene (Doijad et al., 2015). The number of *L. monocytogenes* rods reisolated from the biofilm formed on the steel surface ranged from 6.37 to 7.10 log CFU/cm^2^ and for most strains was higher than on the polypropylene. The differences found were not statistically significant (Table 5). Strains 17, 19, and 20 formed biofilm more strongly on the surface of polypropylene than on steel, with the differences not statistically significant (Table 5). The recovery of bacteria from the biofilm on the polypropylene surface ranged from 6.75 to 8.06 log CFU/cm^2^ (Table 5).

**Table 5:** Intensity of biofilm formation by *L. monocytogenes* strains.

Strain	Stainless steel AISI 304 [log CFU/cm^2^]	Polypropylene [log CFU/cm^2^]	*p*-value
1	6.90 (±0.214)^a,b^	7.42 (±0.262)^a,b^	0.99
3	6.92 (±0.503)^a,b^	6.99 (±0.587)^a,b^	1.00
11	7.01 (±0.708)^a,b^	7.39 (±0.322)^a,b^	0.98
13	6.37 (±0.111)^a^	7.00 (±0.178)^a,b^	0.95
17	6.85 (±0.831)^a,b^	6.74 (±0.588)^a,b^	1.00
18	6.81 (±0.339)^a,b^	7.22 (±0.129)^a,b^	0.97
19	7.10 (±0.081)^a,b^	7.02 (±0.252)^a,b^	1.00
20	7.05 (±0.297)^a,b^	7.04 (±0.693)^a,b^	1.00
21	7.00 (±0.189)^a,b^	7.14 (±0.755)^a,b^	0.99
22	7.08 (±0.521)^a,b^	7.81 (±0.371)^b^	0.89

^a,b,cValues marked with different letters differ statistically significant.^

### 3.8 Evaluation of the efficacy of tested disinfectants against *Listeria monocytogenes* biofilm

**Figure 2:**
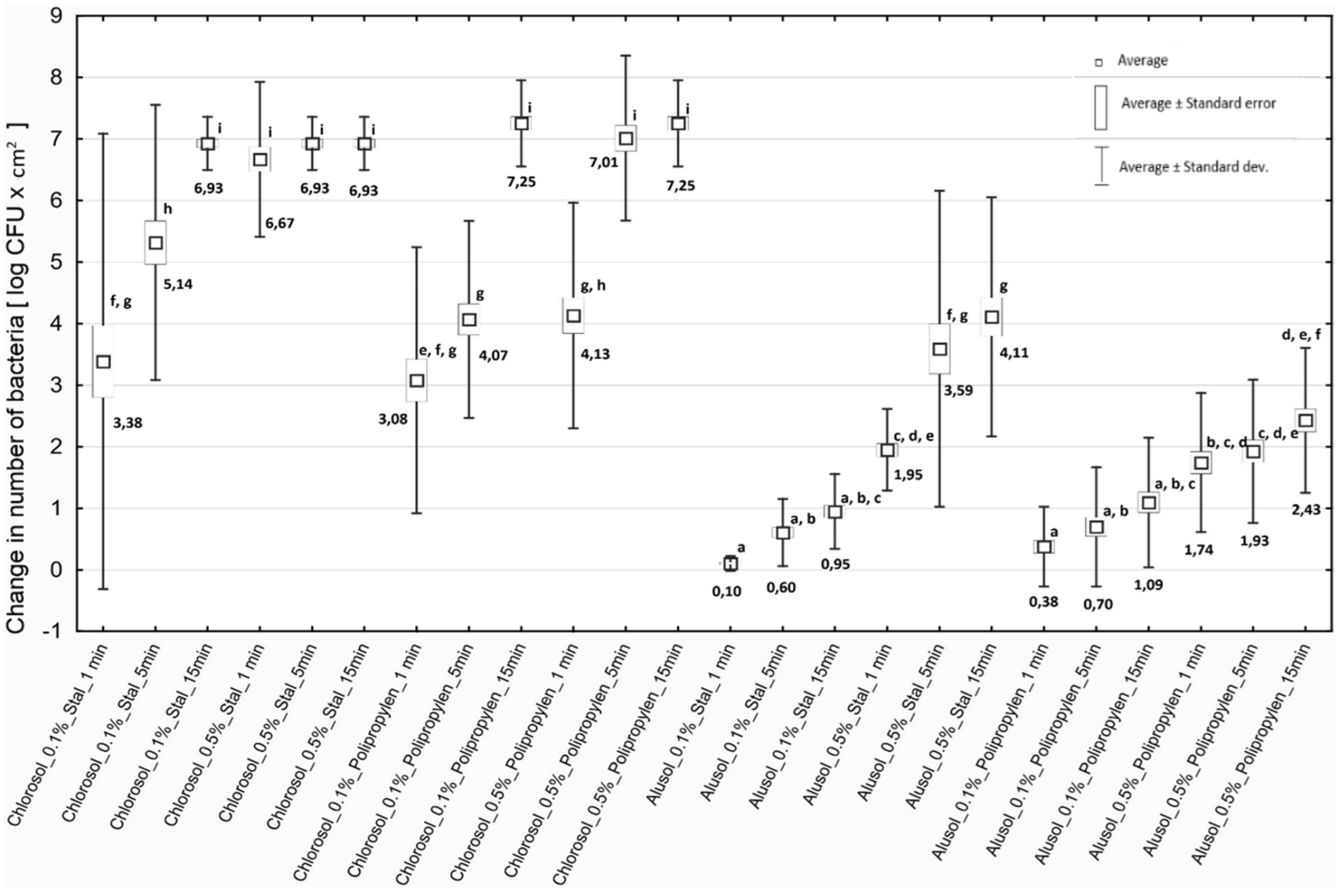
Decreases in bacterial counts depending on the experimental variant used. Steel - stainless steel; Polypropylene—polypropylene.

*Listeria* biofilms contribute to secondary food contamination, posing a threat to public health (Singh et al., 2017). The biofilm structure protects the deeper layers of bacterial cells from disinfectants so it is important to evaluate the effectiveness of available agents. It was shown that both disinfectants tested, regardless of concentration and duration of action, caused a decrease in the number of reisolated bacteria for all strains of *L. monocytogenes* included in the study. The antibiofilm effect depended on the type of agent, its concentration, duration of action and the type of biofilm-covered surface (Figure 2).

### 3.9 Comparison of the effectiveness of disinfectants

Chlorosol was shown to be the disinfectant causing stronger biofilm eradication on each of the tested surfaces. The decreases in the number of bacteria recovered from the biofilm on steel after its use ranged from 3.38 to 6.93 log CFU/cm^2^, and on polypropylene from 3.08 to 7.25 log CFU/cm^2^, and in many cases were equivalent to reducing the number of cells in the biofilm to below the method’s detection threshold (Figure 2). For Alusol, the recorded decreases on steel ranged from 0.10 to 4.11 log CFU/cm^2^, and on polypropylene from 0.38 to 2.43 log CFU/cm^2^ (Figure 2). At a given concentration, duration of action and on the same surface, the decreases in bacterial counts obtained under Chlorosol were statistically significantly higher than those found after application of Alusol (Figure 2).

It was shown that the use of disinfectants at a higher concentration for a time of 1 min produced an effect comparable to their use at a lower concentration with a contact time of 15 min. The exception was the effect of 0.5% Chlorosol on the biofilm formed on polypropylene (Figure 2).

### 3.10 Visual evaluation of the antibiofilm efficacy of the disinfectants used using fluorescence microscopy

**Figure 3:**
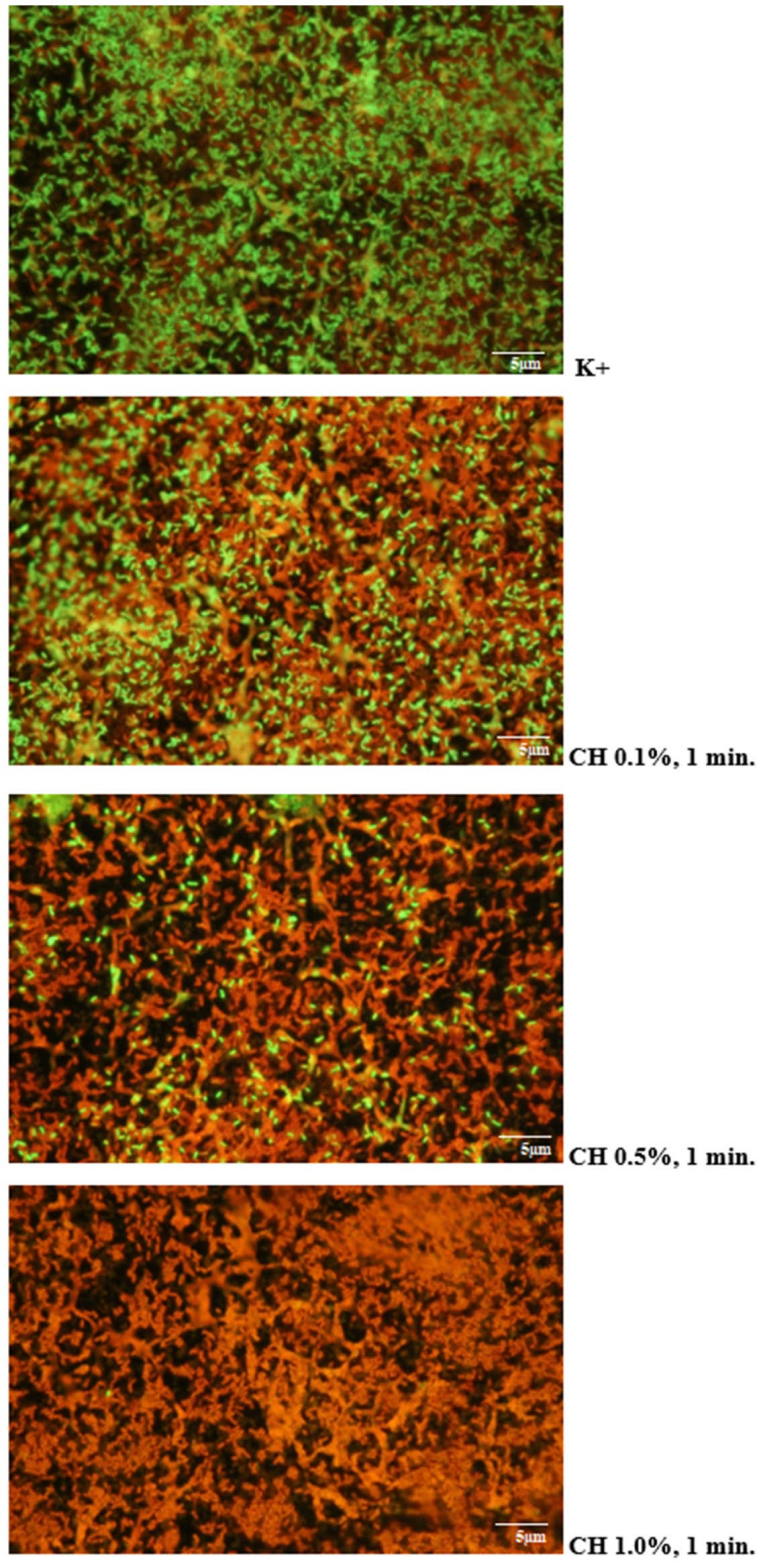
Proportion of living and dead cells in the biofilm layer (CH, chlorosol).

Microscopic images obtained after using the LIVE/DEAD kit confirmed the results of microbiological analyses regarding the effectiveness of the tested disinfectants. It was shown that increasing the concentration of the test agent and extending the duration of its action resulted in an increase in the proportion of dead *L. monocytogenes* cells stained red in the preparation. An example visualization of the effectiveness of Chlorosol against *L. monocytogenes* biofilm on steel is shown in Figure 3.

## 4 Discussion

The intensive development of organic farming generates more and more profits, which gives small and local farmers a chance to develop (Seufert et al., 2017). Among the many legal regulations and principles on which the system of organic farming is based, an important issue is the proper fertilization of crops, in which the use of artificial plant protection products is prohibited, and only fertilizers of natural origin are used. This raises the question of the safety of such fertilizers and the subsequent harvested crops, especially in the context of microbiological risks. The presence of pathogens in the soil and, consequently, in yields from organic crops poses a threat to public health security. Thus, it seems a necessity to evaluate soils and characterize pathogens isolated from the soil. Most food products from organic farming are vegetables and fruits that do not need to be subjected to prior heat treatment. This involves the risk of transmission of many pathogens, including *L. monocytogenes* (Kljujev et al., 2018). In addition, it has been shown (Weller et al., 2016; Harrand et al., 2020) that *L. monocytogenes* can survive at detectable levels in the soil for 128 days after manure application and can potentially contaminate products through soil cultivation or contaminated water. Our own study assessed soil samples collected in southwestern Poland from organic carrot farming. In addition, we evaluated the isolated strains for selected characteristics that affect their virulence. The presence of *L. monocytogenes* rods was found in 27 (10.8%) of the collected soil samples. Genetic similarity analysis (PFGE, “gold standard”) showed 10 genetically different strains that were characterized. Commission Regulation (EU) 2024/2895 of November 20, 2024 on food safety for *L. monocytogenes* sets standards for the pathogen. As of July 2026, a new food safety criterion is to be in effect that *L. monocytogenes* cannot be detected in a 25 g product, including in food marketed during its shelf life (Commission Regulation (EU) 2024/2895, 2024). Our results indicate the presence of *L. monocytogenes* in soil samples, which could result in contamination of carrots and thus pose a risk to future consumers. A microbiological analysis of lettuce, radishes, carrots and beets in Poland was carried out by Szczech et al. (2018), who showed that the number of mesophilic bacteria, yeasts and molds was comparable in the case of radishes and carrots, both in the organic farming system and conventional ones (total number of mesophilic bacteria at the level of 7.0 log10 CFU × g^−1^ and 6.6 log10 CFU × g^−1^, for radishes and carrots, respectively). In turn, Szymczak et al. (2014) showed that 5.0% of organic parsnips tested were contaminated with *L. monocytogenes*. Kljujev et al. (2018) showed that 25.58% of vegetable samples from conventional farming were contaminated with *Listeria* bacteria, while only one of 43 tested samples taken from the field and greenhouse was positive for *L. monocytogenes* (carrot). The researchers also showed that the highest number of *L. monocytogenes* cells was located in the inner layers of the carrot root (10^5^ cells/mm^3^ of dry root) (Kljujev et al., 2018). Our results, as well as data from other researchers, indicate that *L. monocytogenes* is present in both soil and vegetables, which poses a risk to future consumers. The high level of microbial contamination of vegetables indicates their potential spoilage risk and poor quality. According to the literature, leafy vegetables are considered those with the highest potential for microbial risk (Berger et al., 2010). To reduce the risk of foodborne illnesses, it is important to follow proper food safety practices when growing, processing and preparing food.

Due to the systematically increasing number of patients diagnosed with listeriosis and the identification of multidrug-resistant strains, it is advisable to assess antimicrobial resistance among *L. monocytogenes* isolates from food products (European Food Safety Authority and European Centre for Disease Prevention and Control, 2023). In this study, the antibiotic sensitivity of *L. monocytogenes* strains was also assessed. It was shown that 8 (80.0%) strains were sensitive to all tested antibiotics. Our study showed that strain 11 was resistant to penicillin and co-trimoxazole, and strain 22 was resistant to penicillin. Penicillin resistance among 66.7% of tested strains isolated from samples of milk (and milk products), meat (beef and poultry), and fish was demonstrated by Khan et al. (2014). Also, Jamali et al. (2015) observed resistance to penicillin among 83.7% of strains, and to co-trimoxazole in 88.4% of isolates *L. monocytogenes* from food. These data indicate the need to assess antibiotic susceptibility, especially among *L. monocytogenes* strains isolated from food.

Another aspect addressed in the study was the level of invasiveness of *L. monocytogenes* strains toward HT-29 human colon cancer cells. It was shown that the invasiveness ranged from 0.38% (strain 3) to 23.22% (strain 22). Strain 13 did not have the ability to invade cells of the HT-29 line. In turn, Kuda et al. (2015) showed that the level of adhesion of *L. monocytogenes* to intact cells of the HT-29-Luc line was at the level of 6.7 and 6.1 log CFU/well. Jaradat and Bhunia (2003) showed that all strains had the ability to attach to Caco-2 cells, and the level of invasiveness ranged from 1.8 to 31.4%. Similarly, Van Langendonck et al. (1998) observed that all clinical isolates of *L. monocytogenes* were able to invade Caco-2 cells, although the invasiveness level ranged from 0.6 to 23.0%. In turn, Wiktorczyk-Kapischke et al. (2022) showed that the invasiveness of persistent *L. monocytogenes* strains ranged from 1.07 to 11.21%. Both our results and those of other researchers (Kuda et al., 2015; Wiktorczyk-Kapischke et al., 2022) show that *L. monocytogenes* strains are characterized by varied invasiveness. The ability of *L. monocytogenes* to adhere, invade, and grow in intestinal cells is directly related to the virulence of the pathogen (Wałecka-Zacharska et al., 2013), therefore the assessment of this parameter is important for the characterization of these bacteria.

In this study, the metabolic rate of the tested *L. monocytogenes* strains was also assessed based on MTT reduction. Changes in the metabolic activity of the strains were demonstrated, which was correlated with the duration of the experiment. The metabolic activity factor for the tested strains ranged from 0.748 to 1.117. The MTT test is a popular and frequently used tool in estimating the metabolic activity of living cells (Grela et al., 2018). Slama et al. (2012) showed the highest levels of metabolic activity (MTT reduction) were observed for cells subjected to cold stress over a period of 2 years (Slama et al., 2012).

The level of coaggregation between the tested *L. monocytogenes* strains and *S.* Enteritidis rods ranged from 22.2 to 39.1%. In turn, Skowron et al. (2019) showed that the coaggregation level between *L. monocytogenes* and *S.* Enteritidis ranged from 16.5 to 36.3%. Janković et al. (2012) assessed the ability to coaggregate three potential probiotic strains (*Lactobacillus plantarum*) with three pathogens, i.e., *Salomenella* Typhimurium, *L. monocytogenes* strain EGD and *E. coli* (EHEC). The authors of the study showed that all lactobacilli coagulated with the selected food-borne pathogens tested. The level of coaggregation between *L. plantarum* and *L. monocytogenes* ranged from 6.5 to 39.7%. In our study, the lowest coaggregation value between *L. monocytogenes* and *S.* Enteritidis was 22.2%. Gómez et al. (2016) showed that the highest level of coaggregation between *L. monocytogenes* and *Lactobacillus curvatus* was 69.0%, while the lowest level of coaggregation (53.4%) was recorded between *L. monocytogenes* and *Weissella viridescens*. The use of the coaggregation test is a reliable method for assessing the close Gram et al., 2007interaction between lactic acid rods and pathogenic bacteria, especially those responsible for poisoning and infections in the gastrointestinal tract (Collado et al., 2007; Soleimani, 2010).

The ability to form a biofilm determines the presence of *L. monocytogenes* on various surfaces, including vegetables. The biofilm structure is more difficult to remove and thus may not be removed during the vegetable and fruit washing process, posing a risk to public health protection (Botticella et al., 2013). An important aspect of the research was the assessment of the ability of *L. monocytogenes* strains to form a biofilm on the surface of stainless steel and polypropylene. It was shown that most *L. monocytogenes* strains formed a more durable biofilm on the surface of polypropylene than on stainless steel. In turn, Reis-Teixeira et al. (2017) showed that the number of cells adhering to the surface of stainless steel and glass after 3 h of incubation was at the level of 10^5^–10^6^ CFU/cm^2^ and 10^6^–10^8^ CFU/cm^2^ after 24 h. No further an increase in the number of cells in the biofilm structure, despite extending the incubation to 192 h (Reis-Teixeira et al., 2017). However, de Oliveira et al. (2010) showed that *L. monocytogenes* adheres to stainless steel coupons, and the number of cells in the biofilm structure is 4.89 log CFU/cm^2^ after 3 h of incubation. Many studies confirmed the ability *L. monocytogenes* do rapidly adhere to stainless steel surface (Briandet et al., 1999; Ronner and Wong, 1993; Poimenidou et al., 2016). However, these rods do not tend to form thick biofilms composed of several layers (9 to 12 log CFU x cm^−2^), but rather adhere to surfaces at levels of 4–6 log CFU x cm^−2^ (Gram et al., 2007). However, Poimenidou et al. (2016) found that the type of surface significantly influenced the formation of biofilm by *L. monocytogenes*. Poimenidou et al. (2016) showed that the average cell population in the biofilm structure on polystyrene (5.6 log CFU × cm^−2^) was higher than on stainless steel (4.7 log CFU × cm^−2^), which is consistent with our results.

Currently, the resistance of *L. monocytogenes* to disinfectants is the subject of numerous concerns in the context of the food industry and public health. In this study, the biofilm formed by the tested *L. monocytogenes* rods on the surface of stainless steel and polypropylene was treated with specific disinfectants. Both of these agents were effective against *L. monocytogenes* in the biofilm structure, however, their antibiofilm effect was correlated with the type of surface, concentration and type of preparation, and contact time. Higher effectiveness was demonstrated in the case of Chlorosol. During the microscopic assessment of the effect of disinfectants on the formed biofilm, it was shown that increasing the concentration of the tested agent and extending the duration of its action resulted in an increase in the share of dead *L. monocytogenes* cells. In turn, El-Kest and Marth (1988) showed that a solution of 1 mg/mL of free chlorine within 10 min reduced the number of *L. monocytogenes* in 48-h cultures by a factor of 4.27. Bremer et al. (2002) showed a significantly higher percentage of *L. monocytogenes* cells eliminated in the biofilm structure on stainless steel coupons compared to cells on polyvinyl surfaces. Also Pan et al. (2006) showed greater tolerance to the treatment with chlorine-based agents toward the biofilm formed on the Teflon surface compared to the stainless steel surface. Moreover, Folsom et al. (2006) observed that the adaptation of planktonic cells and their subsequent growth on stainless steel makes biofilms more resistant to the action of chlorine-based agents, regardless of the subtype, biofilm cell density and its morphology. In our own study, a disinfectant whose active substance was an aqueous solution of phosphoric and hydrochloric acid (Alusol) was less effective than sodium hypochlorite. In turn, Skoworn et al. (2018) showed that the most effective disinfectant against *L. monocytogenes* was peracetic acid and hydrogen peroxide (decrease in the number of bacteria at the level of 5.10–6.62 log CFU × cm^−2^ and 5.70–7.39 log CFU × cm-^2^, after 1 and 5 min exposure, respectively). However, Beltrame et al. (2015) showed that treatment with peracetic acid and sodium hypochlorite effectively eliminates *L. monocytogenes* from a polyethylene cutting board used in a food processing plant. This study demonstrated that sodium hypochlorite effectively eliminates *L. monocytogenes* in the form of biofilm from the tested surfaces, especially stainless steel. The opposite result was reported by Krysiński et al. (1992), who showed the lowest antimicrobial activity of a chlorine-based disinfectant. In turn, Chen et al. (2015) found that peracetic acid and sodium hypochlorite were ineffective against the tested microorganisms (*L. monocytogenes*, *S.* typhimurium, *E. coli*) in the biofilm structure on the surface of stainless steel. Data on effective methods for eradicating the *L. monocytogenes* biofilm are crucial to maintaining food safety, especially within food processing plants.

The conducted study had some limitations. Soil samples were collected from only two provinces in Poland, which is a small number of samples in relation to epidemiological studies. The number of genetically different *L. monocytogenes* strains examined was only 10. Therefore, further studies covering a larger range of samples and geographical area are recommended. An important aspect that should also be investigated is vegetables and their microbiological state during storage, as a direct factor affecting consumer safety.

## 5 Conclusion

Organic farming is a sector that is developing very dynamically all over the world, but the most dynamically developing organic farming are located in European countries. Although organic farming offers many benefits, it is crucial to highlight the food safety risks, particularly the presence of pathogens in the soil. This work indicates that organic food may be a source of pathogenic microorganisms, such as *L. monocytogenes*, the presence of which was found in 10.8% of soil samples from fields where organic carrots were grown. The work broadly characterized these strains and demonstrated their resistance to antibiotics, invasiveness toward the HT-29 cell line and the ability to coaggregate and form a biofilm. The presence of *L. monocytogenes* strains with high levels of virulence isolated from soil may pose risks to future consumers and public health associated with outbreaks and therapeutic difficulties for antibiotic-resistant strains. The conducted research may help in the future to introduce standards regulating the safety of organic farming. An important aspect would be to evaluate natural fertilizers for the presence of pathogens to avoid their penetration into the soil.

## Data Availability

The raw data supporting the conclusions of this article will be made available by the authors, without undue reservation.
